# Neuroendocrine Carcinomas with Atypical Proliferation Index and Clinical Behavior: A Systematic Review

**DOI:** 10.3390/cancers13061247

**Published:** 2021-03-12

**Authors:** Tiziana Feola, Roberta Centello, Franz Sesti, Giulia Puliani, Monica Verrico, Valentina Di Vito, Cira Di Gioia, Oreste Bagni, Andrea Lenzi, Andrea M. Isidori, Elisa Giannetta, Antongiulio Faggiano

**Affiliations:** 1 Department of Experimental Medicine, “Sapienza” University of Rome, 00161 Rome, Italy; tiziana.feola@uniroma1.it (T.F.); roberta.centello@uniroma1.it (R.C.); franz.sesti@uniroma1.it (F.S.); giulia.puliani@uniroma1.it (G.P.); valentina.divito@uniroma1.it (V.D.V.); andrea.lenzi@uniroma1.it (A.L.); andrea.isidori@uniroma1.it (A.M.I.); elisa.giannetta@uniroma1.it (E.G.); 2 Neuroendocrinology, Neuromed Institute, IRCCS, 86077 Pozzilli (IS), Italy; 3 Oncological Endocrinology Unit, Regina Elena National Cancer Institute, IRCCS, 00144 Rome, Italy; 4 Department of Radiological, Oncological and Pathological Sciences, “Sapienza” University of Rome, 00161 Rome, Italy; monica.verrico@uniroma1.it (M.V.); cira.digioia@uniroma1.it (C.D.G.); 5 Radiology Unit, “Santa Maria Goretti” Hospital, 04100 Latina, Italy; oreste.bagni@uniroma1.it; 6 Endocrinology Unit, Department of Clinical and Molecular Medicine, Sant’Andrea Hospital, Sapienza University of Rome, 00189 Rome, Italy

**Keywords:** neuroendocrine neoplasm, neuroendocrine carcinoma, Ki67 labeling index, Ki67 proliferation index

## Abstract

**Simple Summary:**

Neuroendocrine carcinomas (NECs) are generally highly proliferative and clinically aggressive neuroendocrine neoplasms, but recent literature data suggested that NECs could be further subdivided into two prognostic distinct categories based on the Ki67 labeling index (LI) cut-off of 55%. However, no clear indication on the clinical management and the specific treatment protocol of NECs with a low Ki67 LI are available. We performed a systematic review of the literature to explore the clinicopathological features and the treatment response according to Ki67 LI cut-off in NECs, which is a “hot topic” in neuroendocrinology. Using evidence from 8 studies, for a total of 268 NEC affected patients, the systematic review showed that NECs with a low Ki67 LI had a better prognosis than the subgroup with higher Ki67 LI but worse than G3 neuroendocrine tumors suggesting that NECs are a heterogeneous disease for the pathology findings, the clinical behavior and the treatment response.

**Abstract:**

Background: Highly proliferative (G3) neuroendocrine neoplasms are divided into well differentiated tumors (NETs) and poorly differentiated carcinomas (NECs), based on the morphological appearance. This systematic review aims to evaluate the clinicopathological features and the treatment response of the NEC subgroup with a Ki67 labeling index (LI) < 55%. Methods: A literature search was performed using MEDLINE, Cochrane Library, and Scopus between December 2019 and April 2020, last update in October 2020. We included studies reporting data on the clinicopathological characteristics, survival, and/or therapy efficacy of patients with NECs, in which the Ki67 LI was specified. Results: 8 papers were included, on a total of 268 NEC affected patients. NECs with a Ki67 LI < 55% have been reported in patients of both sexes, mainly of sixth decade, pancreatic origin, and large-cell morphology. The prevalent treatment choice was chemotherapy, followed by surgery and, in only one study, peptide receptor radionuclide therapy. The subgroup of patients with NEC with a Ki67 LI < 55% showed longer overall survival and progression free survival and higher response rates than the subgroup of patients with a tumor with higher Ki67 LI (≥55%). Conclusions: NECs are heterogeneous tumors. The subgroup with a Ki67 LI < 55% has a better prognosis and should be treated and monitored differently from NECs with a Ki67 LI ≥ 55%.

## 1. Introduction

**Figure 1:**
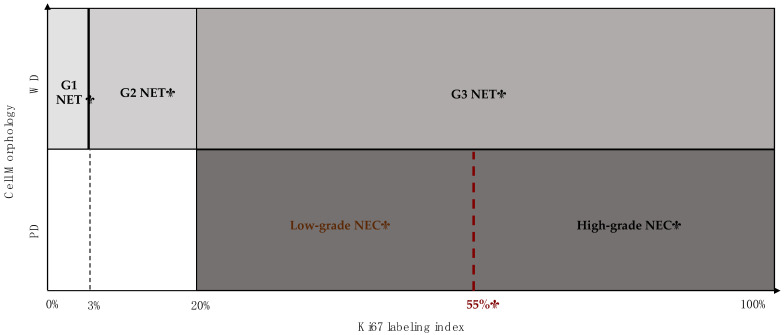
Current World Health Organization (WHO) gastroenteropancreatic neuroendocrine neoplasms (GEP-NENs) classification based on both cell morphology and Ki67 labelling index (LI). G1 neuroendocrine tumor (NET): a well-differentiated (WD) tumor characterized by a mitotic count <2/10 high power fields (HPFs) and/or a Ki67 LI <3%; G2 NET: a WD tumor characterized by a mitotic count 2–20/10 HPFs and/or a Ki67 LI 3–20%; G3 NET: a WD tumor characterized by a mitotic count >20/10 HPFs and/or a Ki67 LI >20%; G3 neuroendocrine carcinoma (NEC): a poorly-differentiated (PD) tumor characterized by a mitotic count >20/10 HPFs and/or a Ki67 LI >20%. As recent literature data suggested, NECs could be further subdivided into two distinct prognostic categories based on the Ki67 LI cut-off of 55%.

Neuroendocrine neoplasms (NENs) are a heterogeneous group of neoplasms arising from the neuroendocrine system, expressing markers of neuroendocrine differentiation as well as hormones and tissue-specific transcription factors [1]. The 2010 World Health Organization (WHO) classification of gastroenteropancreatic (GEP)-NENs divided NENs on the basis of the mitotic count and/or the Ki67 labeling index (LI) in low-intermediate grade (G1-G2) and well differentiated (WD) morphology forms, named “neuroendocrine tumors” (NETs), and high grade (G3) and poorly differentiated (PD) morphology ones, named “neuroendocrine carcinomas” (NECs). According to this classification, G1 NETs are characterized by a mitotic count <2/10 high power fields (HPFs) and/or a Ki67 LI <3%; G2 NETs are characterized by a mitotic count 2–20/10 HPFs and/or a Ki67 LI 3-20%; NECs are characterized by a mitotic count >20/10 HPFs and/or a Ki67 LI >20%. Recently, the 2017 and 2019 WHO classifications of NENs introduced the definition of G3 NET, a WD high grade tumor [2,3]. On this basis, G3 NENs are todays divided into NETs and NECs, based on the morphological appearance. NECs include a small cell (SC) type and a large cell (LC) type, which differ for the cytological details (Figure 1 for NENs nomenclature).

NENs of the lung (Lu-NENs) maintained the 2015 WHO classification into four different variants, based on mitotic count, necrosis areas, and local/distant invasion: typical carcinoid (TC) and atypical carcinoid (AC) as low grade tumors; SC lung carcinoma (SCLC) and LC neuroendocrine carcinoma (LCNEC) as high grade tumors [4].

PD-NECs both from GEP and lung origin are highly proliferative and clinically aggressive NENs, generally characterized by a high rate of the Ki67 LI. These tumors well respond to platinum-based chemotherapy, but the tumor response duration is short. Some literature data suggested that PD-NECs could be subdivided into two prognostic distinct categories based on the Ki67 LI cut-off of 55%, with a better prognosis in patients with a tumor with a Ki67 LI <55%, showing an intermediate behavior between the G3 NETs and the typical NECs [5,6]. Limited evidence supports treatment recommendations specific to NECs, most likely secondary to the lack of sufficient patients numbers to conduct large phase II or III clinical trials [7]. While cisplatin or carboplatin-etoposide treatment regimens are a well-defined first-line therapeutic approach, based on therapy responses reported in SCLC studies [8], the combinations of 5-FU, leucovorin and irinotecan (FOLFIRI) and 5-FU, leucovorin and oxaliplatin (FOLFOX) have shown promising results in GEP-NECs as second line therapy [9,10]. Moreover, temozolomide and capecitabine may be used for patients who failed on first-line chemotherapy [11,12]. The peptide receptor radionuclide therapy (PRRT) also has been proposed as an alternative treatment approach for GEP-NECs [13,14], while immunotherapy is promising, but its effectiveness has not been demonstrated yet in GEP-NECs [15]. At now, effective treatment options alternative to platin-based chemotherapy are very limited.

Recently, it has been reported that NECs with relatively low Ki67 LI better respond to standard therapy than NECs with higher Ki67 LI [6]. However, no clear indication on the treatment of NECs with low Ki67 LI are now available.

We present a systematic review of the literature about the clinicopathological features and the treatment response of the NEC subgroup with Ki67 LI <55%, compared with the subgroup of NECs with higher Ki67 LI (≥55%).

## 2. Materials and Methods

A systematic review was performed following a robust methodology based on the Cochrane Collaboration and PRISMA statements [16,17].

A computerized literature search of MEDLINE and the Cochrane Database of Systematic Reviews revealed that there was no previous publication on G3 NECs with low proliferation rate. English-language original articles were independently searched by two authors (T.F. and R.C.) in several databases (MEDLINE, Cochrane Library, and Scopus) between December 2019 and April 2020. The following key words were used for the study search: ((“cancer” OR “carcinoma” OR “neoplasm” OR “malignant” OR “tumor”) AND “neuroendocrine”) OR “NEC” OR “poorly differentiated neuroendocrine carcinoma” AND “Ki67” OR “low proliferative rate” OR “low proliferation” AND (“management” OR “therapy”). Additional articles were identified by hand-searching reference lists of all the articles retrieved to identify potentially relevant studies. An update of the search was conducted in October 2020.

The titles and the abstracts of all the identified articles were independently screened by two reviewers (T.F. and R.C.) to assess their relevance. Reviews, editorials, letters, and animal studies were excluded. Full texts of selected, potentially relevant, papers were further evaluated. Suitability of the studies was defined for the purpose of this review as reporting on the clinical or biological characteristics, treatment, or clinical outcomes of patients with GEP-NEC or Lu-LCNEC. We selected all the studies that met all the following eligibility criteria: (i) randomized-controlled trial, prospective or retrospective studies; (ii) NEC population defined, according to the 2017 WHO criteria, by both poor cell differentiation and high proliferation indices; (ii) assessment of different subgroups of Ki67 LI (<55% and ≥55%); and (iii) data on survival and/or therapy efficacy for each subgroup. Additionally, studies exploring treatment of G3 NET exclusively were excluded, as were those that did not contain individual data for patients with NEC or provided no data on survival and response rate (RR). Any differences of opinion were resolved by discussion and consensus.

Two authors (T.F and R.C.) independently extracted the following data from included publications: first author, year of publication, study design, study populations, type of NEN, age, sex, cell differentiation and Ki67 LI, therapy regimens, RR, median overall survival (OS), and/or progression free survival (PFS).

## 3. Results

### 3.1. Study Selection

**Figure 2:**
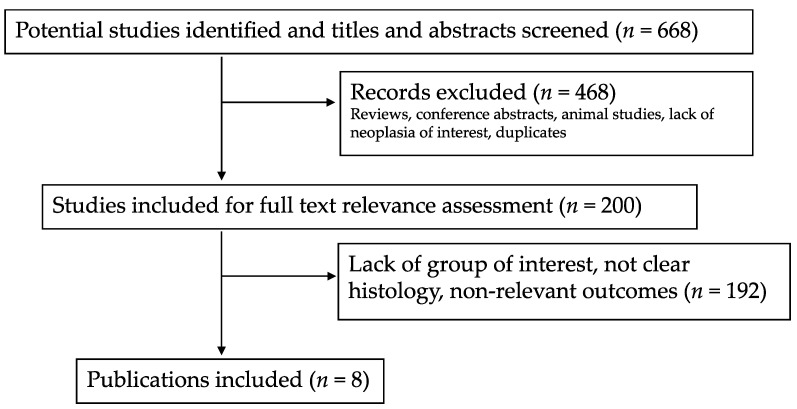
Flow-chart of the literature eligibility assessment process.

Of 668 potentially relevant studies initially identified, 468 were excluded based on the title and abstract screening. The main reasons for exclusion were reviews, conference abstracts, animal studies, duplicates, not clear histology, lack of group of interest (Ki67 LI <55% vs. ≥55%), and non-relevant outcomes. Of the 200 remaining publications, 191 were excluded after full text assessment because they did not meet all the eligibility criteria. All papers in which G3 NECs were not separated from G3 NETs, according to morphological appearance, were excluded, as were those without Ki67 LI subgroups or individual data on Ki67 LI. This process led to the selection of 8 studies. Figure 2 shows the study selection process.

### 3.2. Study Characteristics

The eight studies finally selected were all retrospective studies [6,9,10,18,19,20,21,22] including a total of 268 patients with NECs in which Ki67 LI was specified to distinguish two subcategories: NECs with Ki67 LI <55% and NECs with Ki67 LI ≥55% (Table 1). 

All studies differed according to types of NEN enrolled and treatments received. Specifically, four studies considered both WD G3 NENs and PD-NENs [6,19,21,22], while four studies [9,10,18,20] included only patients presenting the specific subgroup of NECs. NENs derived from different primary sites, the most frequent one being the GEP tract.

As far as NENs treatment is concerned, three studies considered patients treated with different chemotherapy regimens. The studies of Hentic et al. and Hadoux et al. evaluated FOLFIRI and FOLFOX regimens respectively, as second-line treatment but no details were provided about the first-line therapy with platinum-etoposide according to the Ki67 LI [9,10]. The study of Milione et al. considered patients treated with chemotherapy: platinum-etoposide (*n* = 59), other platinum-based regimen (*n* = 31), non-platinum-based chemotherapy (*n* = 12), and other non-cytotoxic therapy (*n* = 8), but unfortunately when the authors stratified the tumors according to the Ki67 LI, they did not provide specific data on the chemotherapy regimen [6]. In four studies, chemotherapy was used combined with other treatment strategies (radiotherapy and/or surgery), specific data on use and outcome of standard first line therapy cannot be extracted from these studies [18,20,21,22]. One study evaluated PRRT efficacy [19].

### 3.3. Clinicopathological Features of Patients with NEC with Ki67 LI <55%

Among all the subjects with NEC, 112 with a Ki67 LI <55% were identified vs. 156 with a Ki67 LI ≥55%, reported in this review as control group. Table 2 summarizes the main demographic and pathological characteristic.

Three studies [9,20,21] reported on patients’ age: the median age was 51 years (range 41.3–52.6) vs. 64.6 (53.4–71) in the subgroup with Ki67 LI ≥55%. Four studies [6,9,20,21] reported on gender: males were 26/49 (53%) among patients with a Ki67 LI <55% NEC, while 59/96 (61.4%) among those with a Ki67 LI ≥55%.

Data about primary NEC site for each Ki67 LI-based subgroup could be extracted from five studies [6,9,18,21,22] (*n* = 189). Pancreas was the prevalent site: among the 62 tumors with a Ki67 LI <55%, 32 had a pNEC (50.8%), whereas 48 out of 127 tumors with a Ki67 LI ≥55% had a pNEC (37.8%). The other GEP primary sites were colon-rectum (*n* = 8, 12.9% vs. *n* = 35, 27.5%), small bowel (*n* = 7, 11.1% vs. *n* = 13, 10.2%), stomach (*n* = 6, 9.7% vs. *n* = 17, 13.4%), liver (*n* = 3, 4.8% vs. *n* = 2, 1.6%), and esophagus (*n* = 1, 1.6% vs. *n* = 4, 3.1%). The remaining GEP cases with Ki67 LI ≥55% were in gallbladder (*n* = 2, 1.6%) and anus (*n* = 1, 0.8%) no Ki67 LI <55% NECs were observed in these sites. Carlsen at al. enrolled only G3 GEP-NECs (44 with Ki67 LI <55% and 11 with Ki67 LI ≥55%), but no further details on primary site were provided. In the study of Hadoux et al., a heterogeneous population of G3 NECs (12 GEP, 4 thoracic, 2 others, 2 unknown) were included. Anyway, when the authors stratified the results according to the Ki67 LI, the site of the tumor was not specified.

Regarding Lu-NENs, in the study of Hermans et al., only 11 patients were included: 6 of them were affected by a NEC with a Ki67 LI <55%, while 5 with a Ki67 LI ≥55% [20].

Four studies reported on morphology of the different subgroups of patients [9,20,21,22] (*n*  = 37): among 12 GEP-NECs with a Ki67 LI <55%, 8 were LC carcinomas (66.7%) while 4 had a SC morphology (33.3%); instead, in the subgroup with a Ki67 LI ≥55%, there were 6/14 GEP-NECs with a LC morphology (42.8%) and 8/14 cases with a SC morphology (57.14%). We only considered lung NECs with a LC morphology (*n* = 11, 100%).

As far as survival and efficacy data is concerned, median OS ranged from 13 months of Basturk et al., to 24.5 months of Milione et al. In the former, there was no survival difference between NEC with Ki67 LI <55% (13 months) vs. ≥55% (16 months) [18]. In the latter, one of the largest studies on pNEC [6], the median OS was the best for G3 NETs (43.6 months), intermediate for NECs with a Ki67 LI ranging 21–54% (24.5 months), and lower for NECs with a Ki67 LI ≥55% (5 months) [6]. The 55% Ki67 LI cut-off was demonstrated as an independent prognostic factor for PD-GEP-NENs [6].

According to the study of Milione et al., PFS and OS differed significantly between patients with different morphology and LI (OS: 44 months vs. 22 months vs. 9 months, PFS: 19 months vs. 11 months vs. 4 months for NETs G3, NECs with Ki67 LI <55%, NECs with Ki67 LI ≥55%, respectively). This was the largest study assessing the outcomes of patients with advanced high-grade GEP-NEN after PRRT [19].

In the study of Hentic et al., in which FOLFIRI regimen was used as second-line therapy after platinum-etoposide in GEP-NECs, the median OS was longer for the subgroup with a Ki67 LI <55% (19.5 vs. 16 months), while the median PFS did not vary between subgroups (4 months) [9]. A longer median PFS characterized the Ki67 LI <55% pNEC compared with the Ki67 LI ≥55% NECs (13 vs. 8 months) in the study of Merola et al., in which a radical intended surgery was proposed for very highly selected stage IV G3 GEP-NENs, with a LCNEC or a G3 NET histopathology [22].

In the study of Hadoux et al., including both GEP and Lu-NENs, there was no difference in terms of RRs according to the 55% Ki67 LI-cutoff, but a longer OS (19.5 months vs. 8.5 months) and PFS (6.2 months vs. 3.6 months) was observed for patients with Ki67 LI <55% [10].

Regarding only Lu-NECs, a prolonged OS (17 months vs. 5 months) and PFS (12 months vs. 3.5 months) was observed in patients with Lu-LCNECs with solitary brain metastases and a Ki67 LI ≤40%, suggesting a prognostic role of the proliferative index [20]. Table 3 summarizes the results on treatment response and outcomes in patients with NEC and low Ki67 LI vs. high Ki67 LI.

## 4. Discussion

We performed a systematic review of the literature to explore the clinicopathological features and the treatment response according to the Ki67 LI cut-off in NECs. A further subclassification of NECs based on this marker is a still debated topic in neuroendocrinology [5]. The current clinical guidelines do not provide specific treatment algorithms for NECs with a low proliferative rate [23]. The most recent WHO classification is designed to guide NENs treatment and prognosis, but unfortunately, because of heterogeneity of these neoplasms, physicians are challenged to treat patients with tumors that cannot be defined to any of the known NEN subtypes [24]. Literature data suggest that the NECs with a low Ki67 LI respond differently to the standard therapy of NECs, showing intermediate features between the G3 NETs and the typical NECs [6]. We found that PD-NECs with a Ki67 LI <55% affected mainly patients of the sixth decade and both sexes, most of them of pancreatic origin with a predominance of LC morphology. The prevalent treatment choice was chemotherapy, followed by surgery with or without chemo- and radiotherapy and only one study evaluated PRRT [19]. The latter study demonstrated that PRRT could be effective in G3 GEP-NEN patients. Most studies showed longer OS and PFS and higher RRs in this category of patients comparing to PD-NECs with higher Ki67 LI, confirming that the Ki67 LI could be a prognostic factor helpful to guide patient management together with the cell morphology. Moreover, in the two studies that evaluated the G3 NETs together with the NECs with a Ki67 LI <55%, this latter had a worse prognosis, confirming that it represents an intermediate category between the G3 NETs and the G3 NECs with a Ki67 LI ≥55%. A recent meta-analysis on the second-line treatment for patients with advanced extra-pulmonary NECs found that studies with a higher proportion of patients with a Ki67 LI >55% had lower RR and shorter OS [25]. Indeed, the relevance of Ki67 LI in NENs has long been reflected in the GEP-NENs classification system, and is also known to be prognostic in the Nordic NEC study, in which a poorer RR to platinum-based chemotherapy in patients with a Ki67 LI <55% compared to patients with a Ki67 LI ≥55% was observed [26]. Otherwise, a recently published retrospective observational French study evaluating platinum- and fluorouracil-based chemotherapy effect on survival in resected GEP-NECs, assessed the role of the Ki67 LI as a prognostic factor and did not find any impact on OS considering 55%, 70%, and 80% threshold while stated that the Ki67 LI ≥80% had a negative prognostic impact on disease-free survival [27].

For what Lu-NENs classification is concerned, the current WHO guidelines do not include Ki67 LI due to some overlap of cut-off thresholds among different tumors [28]. However, the Ki67 LI has been shown to be ≤20% for low- to intermediate-grade pulmonary NETs and >40% for high-grade tumors [29]. A new proposal for a diagnostic algorithm is emerging for Lu-NEN that is, just as for the GEP-NENs, an integration of morphology (necrosis and mitoses) and proliferation (Ki67 LI), aimed at identifying a three-tiers grading system: Lu-NET G1, Lu-NET G2, and Lu-NET G3 [30]. The Ki67 LI effectively separates carcinoids from SCLC and could help for the clinical management and the therapeutic decision-making process of metastatic disease [7]. In the study of Hermans et al., patients with Lu-LCNECs with a solitary brain metastasis and N1 or N0 disease showed in most of the cases a Ki67 LI ≤40% with a prolonged survival compared to patients with a tumor with higher Ki67 LI. Nine of eleven patients were treated with definitive therapy (resection or stereotactic radiotherapy) for both primary and metastatic lesions, instead of standard treatment for stage IV LCNEC with palliative chemotherapy. This study suggests that stage IV Lu-LCNEC is a biological heterogeneous disease and that in some selected cases, a curative treatment could be attempt instead of standard palliative treatment to improve OS. Further prospective studies with larger study populations are needed to confirm these data.

Of note, one Phase II study investigating the efficacy and safety of the second-line FOLFIRI or CAPTEM regimens after failure of the first-line platinum-based chemotherapy in patients with Lu- and GEP-NECs is currently registered at ClinicalTrials.gov (National Clinical Trial identifier NCT03387592). It has been planned to perform a subgroup analysis according to Ki67 LI (21–55% vs. >55%) other than primary tumors site (lung vs. GEP), so it will be hopefully able to carry out useful results for the management of this heterogeneous and rare disease [31].

Our systematic review has some limitations due to the low number of studies on this topic, the heterogeneity of tumor origin and treatment regimens, the small number of patients enrolled in the studies. However, for the best of our knowledge, it is the first systematic review on the “hot topic” of NECs with a low Ki67 LI, according to the latest WHO classification that separated G3 NETs from NECs and indicates the need of further prospective studies with the aim of a better categorization of NECs to improve patients survival outcomes.

## 5. Conclusions

The current systematic review of the literature on NECs with a low Ki67 LI demonstrated that NECs are a rare and heterogeneous disease for the pathology findings, the clinical behavior, and the treatment response. In this context, the 55% cut-off of Ki67 LI could be an important prognostic factor, which is not included in the current NEC WHO classification and consequently, guidelines recommend the same treatment for both low and high Ki67 LI NECs. The systematic review confirmed that NECs with a low Ki67 LI had a better prognosis than the subgroup with higher Ki67 LI but worse than the G3 NETs. Moreover, this subgroup of NECs could benefit more from different treatment strategies that should be validated in prospective, clinical studies.

## Figures and Tables

**Table 1:** Characteristics of the included studies.

First Author (Year) (Reference)	Study Design	Population Object of the Study (*n*)	Primary Site (*n*) Type of Cells (*n*)	Therapy Regimen	Summary
O. Hentic, et al. (2012) [9]	R	GEP-NEC (19)	- Pancreas (10) - Liver (6) - Anus (2) - Pelvic (1) SC (11) LC (6) NA (2)	FOLFIRI as second line after platinum-etoposide	FOLFIRI may be an efficient second-line tx in patients with NECs who are in good condition after failure of platinum-based tx/etoposide.
O. Basturk et al. (2014) [18]	R	NEC (44)	- Pancreas (44) LC (27) SC (17)	S ± adjuvant CHT (cisplatinum based) ± RTx	PD-NEC of the pancreas is a highly aggressive neoplasm, with frequent metastases and poor survival. There was no survival difference between Ki67 LI <55% NEC patients and Ki67 LI ≥55% ones.
J. Hadoux et al. (2015) [10]	R	NEC (20)	- -GEP (12) - Thoracic (4) - -Other (2) - Unknown (2) SC (7) LC (12) NA (1)	FOLFOX as second line after platinum-etoposide	FOLFOX regimen may be an effective second-line tx in NEC patients after platinum-based first-line treatment. There was no difference in terms of RRs according to the 55% Ki67 LI-cutoff. A longer PFS and OS was observed for patients with Ki67 LI <55% NECs.
M. Milione et al. (2017) [6]	R	G3 GEP-NEN (136) -G3 NET (24) -NEC (112)	- Pancreas (22) - Stomach (23) - Esophagus (5) - Duodenum (5) - -Ileum-cecum (13) - -Colon-rectum (42) - -Gallbladder (2) NA	Platinum-etoposide (*n* = 59), Other platinum-based CHT (*n* = 31), Non-platinum-based CHT (*n* = 12), Other non-cytotoxic tx (*n* = 8)	Median OS was best for G3 NET, intermediate for NEC with a Ki67 LI 21–54% and lower for NEC cases with a Ki67 LI ≥55%. The 55% Ki67 LI cut-off is an independent prognostic factor for PD-GEP-NENs.
E. A. Carlsen et al. (2019) [19]	R	G3 GEP-NEN (149) -G3 NET (60) -NEC (62) -NA (27)	- -Pancreas (89) - Unknown (26) - -Other (34) NA	PRRT	PRRT can be effective in high-grade GEP-NEN patients. PFS and OS differed significantly in patients according to differentiation and proliferation.
B. C. M. Hermans et al. (2019) [20]	R	LCNEC with a solitary brain metastasis (11)	- Lung (11) LC (11)	9/11: definitive tx (S ± SRT ± CHT) 2/11: metastasectomy ± SRT brain	Stage IV LCNEC with a solitary brain metastasis and N0/N1 disease show in the majority of cases Ki67 LI ≤40% and prolonged survival, distinguishing them from general LCNEC.
H. Kim et al. (2020) [21]	R	NEN (82) -G1 NET (20) -G2 NET (47) -G3 NET (8) -NEC (7)	- Pancreas (82) SC (4) LC (3)	S ± CHT	Histological features supporting the diagnosis of pNECs over G3 pNETs were the absence of a low-grade pNET component, the presence of diffuse marked nuclear atypia, solid growth pattern, frequent apoptosis, and markedly increased proliferative activity. No statistical analysis was performed between the two subgroups (Ki67 LI <55% vs. Ki67 LI ≥55%)
E. Merola et al. (2020) [22]	R	G3 GEP-NEN (15) -G3 NET (7) -NEC (6) -MiNEN (2)	- Pancreas (9) - Colorectal (2) - Gastro-esophageal (1) - Ileum (1) - Appendix (2) LC (6)	S + CHT (cisplatinum/etoposide)	Radical intended surgery may be considered for very highly selected stage IV GEP-NENs G3, with a LCNEC or a NET G3 histopathology. No statistical analysis was performed between the two subgroups (Ki67 LI <55% vs. Ki67 LI ≥55%).

CHT, chemotherapy; CRS, cytoreductive surgery; FOLFIRI, folinic acid, 5-fluorouracil and irinotecan; FOLFOX, 5-fluorouracil and oxaliplatin; G1, grade 1; G2, grade 2; G3, grade 3; GEP, gastroenteropancreatic; large cell neuroendocrine carcinoma; MiNEN: mixed neuroendocrine-non-neuroendocrine neoplasm; mo, months; NA, not available; NEC, neuroendocrine carcinoma; NEN, neuroendocrine neoplasm; NET, neuroendocrine tumor; OS, overall survival; PD, poorly differentiated; PFS, progression-free survival; pNEC, pancreatic neuroendocrine carcinoma; pNET, pancreatic neuroendocrine tumor; PRRT, peptide receptor radionuclide therapy; px, patients; R, retrospective; RTx, radiotherapy; RR, response rate; S, surgery; SRT, stereotactic radiotherapy; SSA, somatostatin analog; TMZ, temozolomide; tx, therapy.

**Table 2:** Demographic and pathological features of patients with neuroendocrine carcinoma (NEC) and low Ki67 labeling index (LI) vs. high Ki67 LI.

NEC Features	Ki67 LI < 55%	Ki67 LI ≥ 55%
*n*/tot (%)	112/268 (41.8)	156/268 (58.2)
Median age, yrs (range)	51 (41.3–52.6)	64.6 (53.4–71)
Sex, *n*	49	96
Male:Female	26:23	59:37
Primary site, *n* (%)	62	127
Pancreas	32 (51.6)	48 (37.8)
Colon-rectum	8 (12.9)	35 (27.5)
Small bowel	6 (11.1)	13 (10.2)
Stomach	6 (9.7)	17 (13.4)
Lung	6 (9.7)	5 (3.9)
Liver	3 (4.8)	2 (1.6)
Esophagus	1 (1.6)	4 (3.1)
Gallbladder	−	2 (1.6)
Anus	−	1 (0.78)
Type of cells, *n* (%)		
LC GEP-NECs	8/12 (66.7)	6/14 (42.8%)
SC GEP-NECs	4/12 (33.3)	8/14 (57.1%)
LC Lung-NECs	6/11 (54.5)	5/11 (45.5%)

LC, large cell; LI, labeling index; GEP, gastroenteropancreatic; mo, months; *n*, number; NEC, neuroendocrine carcinoma; OS, overall survival; PFS, progression-free survival; SC, small cell; yrs, years.

**Table 3:** Treatment response and outcomes in patients with neuroendocrine carcinoma (NEC) and low Ki67 labeling index (LI) vs. high Ki67 LI.

Authors (Year) (Reference)	NEC Subgroups for Ki67 LI					
	Ki67 LI < 55%			Ki67 LI ≥ 55%		
	RR, %	Median OS, mo (Range)	Median PFS, mo (Range)	RR, %	Median OS, mo (Range)	Median PFS, mo (Range)
O. Hentic et al. (2012) [9]	6 DC 4 PD (2 px still alive, >30 mo)	19.5 (12–28)	4 (1–8)	3 DC 2 PD	16 (11–26)	4 (2–7)
O. Basturk et al. (2014) [18]	NA	13 (6–20)	NA	NA	16 (6–24)	NA
J. Hadoux et al. (2015) [10]	NA	19.5	6.2	NA	8.5	3.6
M. Milione et al. (2017) [6]	NA	24.5 (16.9–29.0)	NA	NA	5.3 (3.3–8.9)	NA
E. A. Carlsen et al. (2019) [19]	CR: 3 PR: 41 SD: 31 PD: 26	22 (16.0–28.0)	11 (5.4–16.6)	CR: 0 PR: 45 SD: 9 PD: 45	9 (1.6–16.4)	4 (0.8–7.2)
B. C. M. Hermans et al. (2019) [20]	NA	17 (11–23) (2 px still alive, >5 yrs)	12 (5–51)	NA	5 (0.7–9.3)	3.5 (2–4)
H. Kim et al. (2020) [21]	NA	15 (4–60)	8 (3–15)	NA	8 (2–17)	5.5 (2–17)
E. Merola et al. (2020) [22]	NA	23 (1 px still alive)	13	NA	14.5 (8–35) *	8 (5–16)

CR, complete response; DC, disease control; LI, labeling index; mo, months; *n*, number; NA, not available; OS, overall survival; PD, progressive disease; PFS, progression-free survival; PR, partial response; px, patient(s); RR, response rate; SD, stable disease; yrs, years; * Data of 4 pts out 5.

## Data Availability

No new data were created or analyzed in this study. Data sharing is not applicable to this article.
